# Scoping review of therapeutic approaches among individuals with secondary exercise addiction

**DOI:** 10.3389/fpsyt.2026.1780217

**Published:** 2026-04-28

**Authors:** Magdalena Jaroch-Lidzbarska, David Alarcón, Henryk Olszewski, Dominika Wilczyńska

**Affiliations:** 1 Department of Physical Culture, Gdansk University of Physical Education and Sport, Gdańsk, Poland; 2 Faculty of Social and Humanities, WSB Merito University, Gdańsk, Poland; 3 Department of Social Anthropology, Universidad Pablo de Olavide, Sevilla, Spain; 4 Department of Psychology and Developmental Psychopathology, University of Gdansk, Gdańsk, Poland

**Keywords:** effective therapeutic interventions, exercise addiction level data, research gap, secondary exercise addiction, therapeutic approaches

## Abstract

Secondary exercise addiction shows high comorbidity with eating and body image disorders. Despite its substantial impact on physical and mental health and daily functioning, evidence on effective therapeutic interventions remains limited. The aim of this scoping review was to identify and describe therapeutic interventions applied to adult individuals with secondary exercise addiction. This review followed the PRISMA Sc-R guidelines and covered the years 2002–2024. Ultimately, five studies were included (four randomized controlled trials and one quasi-experimental study). Three studies applied psychotherapeutic interventions based on cognitive-behavioral models (Cognitive Behavioral Therapy, Lifestyle, Exercise, Attitudes, and Relationships Program, Physical Exercise and Dietary Therapy), while two integrated physical or nutritional components. A secondary analysis published in 2024 based on the LEAP trial dataset was identified but not treated as an independent study to avoid duplication. EBSCOhost, Web of Science, PubMed, and Google Scholar were searched from January to May 2025 using terms related to exercise addiction, exercise abuse, psychotherapy, intervention, and treatment. English-language studies were eligible if they described an intervention with at least one treated group with pre- and post-test measures; the participants of the study were adult patients suffering from eating disorders and exercise addiction (the therapy programs involved one inpatient and four outpatient treatments) and therapeutic intervention was carried out with outcomes based on exercise addiction level data. Four out of five included studies reported improvements in variables related to compulsivity, although these did not always imply a reduction in the amount of exercise, indicating that qualitative changes may be more relevant. Longer interventions showed more consistent effects, but even brief treatments generated positive changes in non-clinical populations. The examination of the research revealed a gap in studies addressing interventions for those with secondary exercise addiction, especially highlighting the need for randomized controlled trials (RCTs) with proper randomization methods.

## Introduction

Exercise addiction is classified as a behavioral problem that can have negative consequences, affecting daily life and optimal performance. Compulsive exercise is associated with highly driven and rigid urge to exercise, synchronic with a perceived inability to stop exercising, despite awareness of all harm risk resulting from continuing the exercise (1). The conceptualizations of maladaptive exercise include both quantitative components (frequency, volume, duration) and qualitative aspects associated with exercise-related cognitions (obsession, compulsivity, rigidity) (2).

Exercise dependence in its primary form entails pathological exercise behaviour which is driven solely for psychological gratification coming from exercise behaviour alone (3), while in its secondary form, it shows high comorbidity with eating and body image disorders (4) and is often used as a means to regulate the distress associated with these conditions (5).

Despite its significant impact on the psychological and physical health of people suffering from primary or secondary exercise addiction, there is limited knowledge about effective treatment methods for this condition. Furthermore, there is little research on this subject in the related literature.

However, adjusted therapeutic guidelines for treating exercise addiction have not yet been developed. One reason for this can be the fact that this form of addiction does not appear in the standard diagnostic manuals currently in use (DSM-5, ICD-10, and even ICD-11). Furthermore, exercise addiction is rare, and individuals who are addicted do not often seek help from healthcare professionals (6).

Prior to providing treatment for exercise addiction, it is inevitable to conduct an accurate diagnosis to draft an appropriate psychotherapeutic plan for the specific needs of the patient.

It is necessary to diagnose co-occurring disorders (e.g., personality, depressive, and/or anxiety disorders) because they must also be treated. Another important issue is, that during treatment of exercise addiction, concentration on the abstinence may not be the goal, but a return to moderate and controlled exercise (7).

An important goal of treatment for people suffering from exercise addiction should be to help them first recognize and then reduce their maladaptive behaviors and exercise cognition. It is necessary to teach the patients how to (re)establish a healthy relationship with exercise to be able to benefit from exercising rather than suffering because of uncontrolled sports undertaking (8).

Adams et al. indicate, that the core of psychotherapeutic interventions in the problem of exercise dependence should include the following steps:

1. identifying and interrupting the compulsory behaviour
2. helping the patient to understand the health benefits and importance of moderation
3. engaging the patient in developing the self-management strategies
4. understanding the patient’s coping strategies with the addiction
5. modifying the patient’s psychological defenses and developing appropriate self-management skills
6. identifying the triggers of the exercise dependence
7. rebuiliding the patient’s coping behaviors and enhancing the support system with the respect to exercise (9).

The above-mentioned guides make it clear that the process of recovery from this addiction can be time-consuming and engaging.

The aim of this scoping review was to identify and describe therapeutic interventions applied to individuals with secondary exercise addiction. The review focused on evaluating the types of interventions used (e.g., cognitive-behavioral, physical, dietary), their reported outcomes on compulsive exercise behaviors, and the methodological quality of the studies. Additionally, we aimed to identify current research gaps and limitations in the existing literature to inform future intervention development.

## Methods

### Eligibility criteria

**Figure 1:**
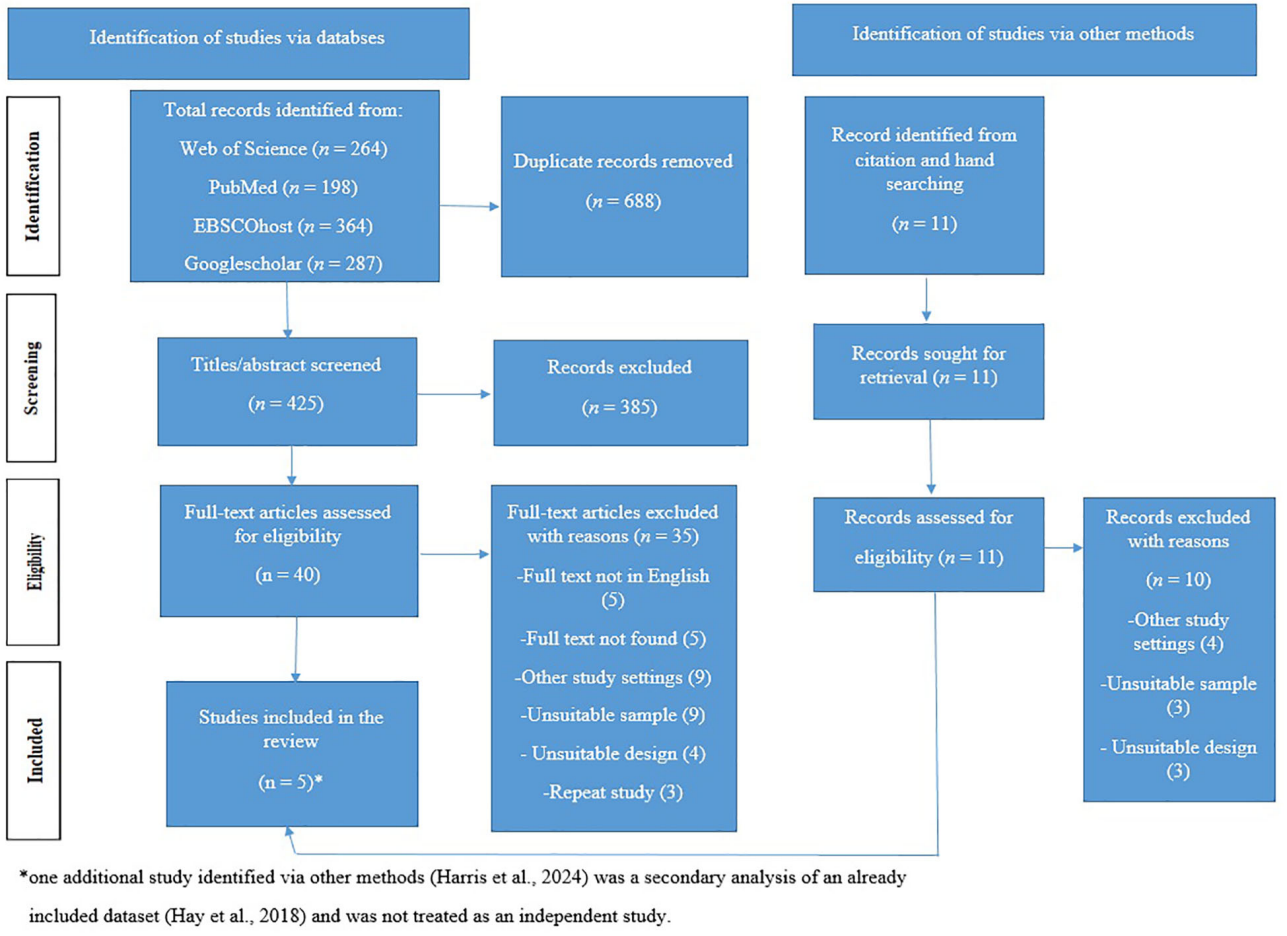
PRISMA-ScR flow diagram (10). PRISMA-ScR: Preferred Reporting Items for Systematic Reviews and Metaanalyses for Scoping Reviews.

The search result is reported with the Preferred Reporting Items for Systematic Reviews and Meta-analyses for Scoping Reviews (PRISMA-ScR), used together with the checklist (10). The details of the search are presented in the PRISMA-ScR flow diagram (**Figure 1**).

Studies were included if they met the following criteria: (a) at least one treated group with pre- and post-intervention measures; (b) adult participants diagnosed with eating disorders and exhibiting compulsive or exercise addiction-related behaviors; and (c) implementation of a therapeutic intervention with outcomes based on exercise-related measures. Only studies published in English were included. Screening was conducted in two stages: (1) title and abstract screening and (2) full-text assessment. Two reviewers independently assessed eligibility, and disagreements were resolved through discussion until consensus was reached.

### Information sources

The aim of this review was to evaluate the effectiveness of therapeutic interventions in individuals with eating disorders and exercise addiction. The search covered studies published between 2002 and 2024.

A systematic search was conducted between January and May 2025 by two reviewers (M.J.-L. and D.W.) using the following electronic databases: EBSCOhost, Web of Science, PubMed, and Google Scholar. In addition, citation tracking and manual searches of reference lists were performed to identify further relevant studies.

### Search strategy

The search strategy combined terms related to exercise addiction and eating disorders with terms related to therapeutic interventions. The following keywords were used: (“exercise addiction” OR “exercise dependence” OR “compulsive exercise” OR “exercise abuse” OR “obsessive exercise”) AND (“psychotherapy” OR “therapy” OR “counselling” OR “intervention” OR “treatment”).

Database-specific adaptations were applied where necessary. For example, in EBSCOhost, an advanced search was conducted using two separate search fields: one for exercise-related terms and one for intervention-related terms.

### Study selection and data extraction

All identified records were imported and duplicates were removed prior to screening. Titles and abstracts were screened for relevance, followed by full-text assessment based on the predefined inclusion criteria.

Data extraction was conducted using a standardized form. The following information was extracted from each study: author, year of publication, study design, sample characteristics, type of intervention, duration of intervention, and outcome measures. Where multiple publications reported data from the same study sample, they were treated as a single dataset to avoid duplication.

## Results

Five studies met the inclusion criteria and were included in the final analysis. These comprised four randomized controlled trials and one quasi-experimental study. An additional study published in 2024 (11) was identified; however, it represented a secondary analysis of data from an already included randomized controlled trial (12) and was therefore not treated as an independent study.

### Study quality assessment

The methodological quality of the included studies, assessed using the Jadad scale, is presented in **Table 1**. Two studies were rated as “adequate” (score = 2), while three were classified as “low” quality (score = 0).

**Table 1:** Study quality (own elaboration based on the Jadad scale).

Articles	Criteria: (*) yes = 1/no = 0; (**) yes = 1/no = -1					Score of studies	Quality
	The study is described as randomized (*)	The study is described as double-blind (*)	Losses and withdrawals from the study are described (*)	The randomization method is adequate (**)	The double-blind method is adequate (**)		
Hay et al. (12)^1^	1	1	0	1	-1	2	Adequate
Zeeck et al. (13)	1	0	1	-1	-1	0	Low
Calogero and Pedrotty (14)	0	0	0	-1	-1	0	Low
Mathisen et al. (15)	1	0	1	1	-1	2	Adequate
Sundgot-Borgen et al. (16)	1	0	1	-1	-1	0	Low

^1^ Secondary analysis of this trial was reported in Harris et al. (11).

The studies rated as adequate (12, 15) reported appropriate randomization procedures and provided information on withdrawals. However, none of the included studies fulfilled the criteria for adequate double-blinding, which lowered the overall quality scores. Studies classified as low quality (13, 14, 16) either lacked sufficient detail on randomization procedures or did not report participant attrition.

### Characteristics of included studies and interventions

A detailed overview of study characteristics, interventions, and outcomes is provided in **Table 2**. The included studies varied in design, intervention type, and treatment setting (inpatient vs outpatient).

**Table 2:** Results of the scoping review (own elaboration).

No.	Author and date of publication	Age (years)	Participants	Intervention program	Program duration	Frequency	Research details
1	Hay et al. (12)	LEAP *M* = 26,1 years; CBT-AN *M* = 28,6 years	n=78 (5% men – LEAP; 5,3% men – CBT-AN)	CBT- AN + LEAP vs CBT-AN	8–10 months	two sessions of 50 minutes a week for four weeks and after one session of 50 minutes a week	CBT-AN and LEAP added to CBT-AN resulted in improved attitudes and beliefs but there were no significant differences between treatment groups (CBT-AN vs CBT-AN + LEAP) toward exercise
2	Zeeck et al. (13)	Sport therapy group *M* = 24,3 years; Control group *M* = 27,2 years	n=23 (not specified)	sport therapy program	3 months	one introductory session and then one 2-hour group session a week	Avoidance and rule driven behavior was significantly more reduced after the sport therapy program compared to the CG in the intention to treat (ITT) as well as the completer analysis; no significant difference in the reduction of exercise quantity
3	Calogero and Pedrotty (14)	Exercise group *M* = 22.49; Control group *M* = 23.14	n=254 (no men)	exercise program	not specified	one session of 60 minutes four times per week	women in the exercise group demonstrated significantly reduced obligatory attitudes toward exercise compared to the control group
4	Mathisen et al. (15)	18–40 years PED-t *M* = 28.2 years; CBT *M* = 27,7 years; Control group *M* = 26,5 years	n= 187 (no men)	PED-t or CBT	16 weeks	1–2 sessions a week (length of session not specified)	both therapies resulted in significant improvements in compulsive exercise reduction, a change not found in the control group
5	Sundgot-Borgen et al. (16)	18–29 years CBT *M* = 22 years; Exercise *M* = 23 years; Nutrition *M* = 22 years	n= 64 (no men)	physical exercise program, CBT and nutritional advice	16 weeks	one 2-hour group session a week	Nutritional counselling did not prove more effective than CBT. Physical exercise appeared more effective than CBT in reducing pursuit of thinness; change in body composition; aerobic fitness; and frequency of bingeing, purging, and laxative abuse
6	Harris et al. (11) Secondary analysis of Hay et al. (12) dataset	LEAP *M* = 26,1 years; CBT-AN *M* = 28,6 years	n=78 (5% men – LEAP; 5,3% men – CBT-AN)	CBT- AN + LEAP vs CBT-AN	8–10 months	two sessions of 50 minutes a week for four weeks and after one session of 50 minutes a week	CBT-AN and LEAP added to CBT-AN resulted in improved attitudes and beliefs but there were no significant differences between treatment groups (CBT-AN vs CBT-AN + LEAP) toward exercise

M, mean age (years).

n, number of participants.

CBT-AN, cognitive behavioral therapy for anorexia nervosa.

LEAP, Lifestyle, Exercise, and Activity Program.

PED-t, physical exercise and diet therapy.

CG, control group.

ITT, intention-to-treat analysis.

CBT, cognitive behavioral therapy.

Three studies implemented psychotherapeutic interventions based on cognitive-behavioral models (CBT, LEAP, PED-t), while two studies applied interventions focused primarily on behavioral or physical activity components. One study employed a mixed approach combining psychotherapy, dietary, and behavioral management components. A categorization of intervention types is presented in **Table 3**.

**Table 3:** Division of therapeutic intervention applications.

Type of intervention	
Mixed therapy (dietary, psychoterapeutic and behavioral management program)	Behavioral management
Hay et al. (12) CBT- AN + LEAP vs CBT-AN (data also reported in Harris et al., 2024 (11))	Zeeck et al. (13) sport therapy program
Mathisen et al. (15) PED-t or CBT	Calogero and Pedrotty (14) exercise program
Sundgot-Borgen et al. (16) physical exercise program, CBT and nutritional advice	

CBT-AN, Cognitive Behavioral Therapy for Anorexia Nervosa.

LEAP, Lifestyle, Exercise, and Activity Program.

PED-t, Physical Exercise and Diet Therapy.

### Effects of interventions

Four out of five studies reported improvements in variables related to compulsive or maladaptive exercise behaviors. These improvements were primarily observed in qualitative aspects of exercise (e.g., reduced rigidity, improved cognitive control, or changes in attitudes toward exercise), rather than in the overall quantity of physical activity.

Longer and more structured interventions, particularly those based on cognitive-behavioral frameworks, demonstrated more consistent effects. However, even shorter or less intensive interventions were associated with positive changes, especially in non-clinical or mixed populations.

Overall, the findings suggest that therapeutic interventions targeting exercise-related psychopathology may be effective, although the limited number of studies and variability in methodological quality restrict the strength of conclusions.

## Discussion

The objective of our review was to identify the interventions employed in treating individuals who are addicted to exercise and simultaneously suffering from eating disorders (secondary exercise addiction). Additionally, we placed particular emphasis on the assessment tools used with the study participants. Our examination of the research reveals a gap in studies addressing interventions for those with exercise addiction, especially highlighting the need for randomized controlled trials (RCTs) with proper randomization methods. In our review, four studies were RCTs; however, half of them lacked adequate randomization methods (**Table 1**), underscoring the necessity for high-quality research in this field. Future researchers should ensure the use of robust randomization methods, such as computer-generated sequences, along with proper allocation concealment and transparent reporting in line with established guidelines.

The studies analyzed show remarkable heterogeneity in the therapeutic approaches applied to people with secondary exercise addiction, reflecting both the clinical complexity of the disorder and the absence of standardized protocols. Although all studies focus on patients with eating disorders, they differ in methodological design, type of intervention, duration, sample composition, and results. Three studies applied psychotherapeutic interventions based on cognitive-behavioral models (CBT, LEAP, PED-t), while two integrated physical or nutritional components. This diversity suggests the need for integrative approaches. In general, most studies—except Zeeck et al.—reported improvements in variables related to compulsivity, although these did not always imply a reduction in the amount of exercise. While this may appear inconsistent with traditional models of addiction, in which reduction or cessation of the behavior is a primary goal, the context of eating disorders requires a more nuanced interpretation. In this population, exercise often serves complex psychological functions, including emotion regulation, anxiety reduction, and compensation for perceived caloric intake. Accordingly, compulsive exercise should be conceptualized not only in quantitative terms (e.g., frequency or duration), but also in relation to qualitative dimensions, including cognitive rigidity, loss of control, and maladaptive motivations. From this perspective, therapeutic success may be better reflected in qualitative changes—such as increased behavioral flexibility, reduced rigidity, and more adaptive attitudes toward exercise—rather than solely in decreased exercise volume. This interpretation is supported by previous research indicating that treatment effects on compulsive exercise are often modest and that pathological exercise is more accurately characterized by its qualitative features (e.g. rigidity, affect regulation) rather than purely quantitative indices (e.g. frequency or duration) (17, 18). Cognitive-behavioral interventions, including LEAP and PED-t, may facilitate these changes through mechanisms such as cognitive restructuring, improved emotion regulation, and increased awareness of maladaptive exercise patterns.

Longer interventions (Hay et al., Mathisen et al.) showed more consistent effects, but even brief treatments (Calogero&Pedrotty) generated positive changes in non-clinical populations. The limited inclusion of men and the concentration of participants in early adulthood limit the generalizability of the findings. Furthermore, methodological quality was uneven: some studies lack key information on randomization or blinding, which reduces the robustness of their conclusions.

Although the methodological quality of included studies was low according to the Jadad scale, the overall findings should be interpreted with caution rather than dismissed. Importantly, the results were generally consistent across studies, including those with higher quality scores, which supports the robustness of the observed trends. Moreover, given the limited number of available RCTs in this field, these studies still contribute valuable preliminary evidence, highlighting the need for more rigorously designed trials to confirm these findings.

Finally, the results suggest a tension between interventions aimed at cognitive restructuring and those that seek to reintroduce exercise in a controlled manner. In the study by Sundgot-Borgen et al., structured exercise showed greater benefits than CBT in variables such as the pursuit of thinness, highlighting the importance of the context and purpose of exercise in therapeutic efficacy.

In conclusion, interventions based on cognitive-behavioral models appear promising for treating secondary exercise addiction. However, methodological heterogeneity, limited sample diversity, and a lack of standardized measures make it difficult to draw firm conclusions. Future studies with greater population diversity and designs that integrate cognitive, behavioral, and physical components are needed.

Numerous scientific reports have been published on the effectiveness of both behavioral and psychotherapeutic interventions in professionally trained athletes (19), as well as in patients simultaneously struggling with secondary exercise addiction (20). However, these reports pertain to interventions for individual cases and have not been tested in larger cohorts. Research development in this area holds substantial potential for establishing an effective therapeutic protocol for individuals struggling with eating disorders, a population that poses significant clinical challenges for mental health professionals because of the compensatory behaviors that often accompany these conditions, such as compulsive exercising.

## Limitations

The limitations of this review encompass the heterogeneity of study populations, intervention protocols, outcome measures, and assessment tools, in addition to the risk of incomplete literature coverage. Furthermore, the inclusion of only English-language sources may have resulted in the omission of potentially relevant studies. Finally, these results might not apply to men or older people because most participants of the studies were young women.
